# Ethnobotanical study of medicinal plants used in Arjan – Parishan protected area in Fars Province of Iran

**Published:** 2014

**Authors:** Mehdi Dolatkhahi, Ali Dolatkhahi, Javad Bagher Nejad

**Affiliations:** 1 *Department of Biology, Bushehr Branch, Payam Noor University, Bushehr, I. R. Iran*; 2 *Department of Horticultural Science, Faculty of Agriculture, Ferdowsi University of Mashhad, Mashhad, I. R. Iran *; 3 *Section of Agriculture, Management of Kazeroon Jahad Keshavarzi, Fars province, I. R. Iran*

**Keywords:** *Arjan* – *Parishan area*, *Ethnobotany*, *Fars province*, *Medicinal plants*, *Pharmacological*, *Iran*

## Abstract

**Objective** **: **Today, medicinal plants are widely used in remedies for several ailments and improvement of human health because of their pharmaceutical properties. This study aimed to document important useful medicinal plants and their medicinal characteristics for treatment of human ailments in the Arjan ^_^ Parishan protected area in Fars province of Iran during 2010-2012.

**Materials and Methods** **: **Data were obtained using direct interviews with 80 informants particularly those who were more familiar with the herbs and their medicinal properties. Collected plants were recognized and families, genera, and species determined using indispensable references. In this paper, scientific name, local name, parts used, and ways of application and ailments treated using traditional medicinal plant species have been provided.

**Results** **: **We documented 85 plant species belonging to 39 families and 78 genera used for treating ailments. Among which, Asteraceae with 13 species was the most frequently used family and fruits and leaves were the favored parts for local users. Our results indicated that in this area, the highest compliance in the use of plants in treating ailments were related to the intestinal digestive system (40.8%).

**Conclusion** **: **The present study is the first contribution to the ethnobotany of this region. Our results showed that some plants are used for medicinal purposes in this region, either for the same or for different purposes. Generally, the results of the present investigation can be used as a basis for selecting useful medicinal plants and also help to preserve precious information that may otherwise be lost to future generations.

## Introduction

Nowadays, medicinal plants are extensively utilized in traditional medicine for treating ailments (Davidson-Hunt, ‎‎2000 ▶). There is an increasing interest in public for consumption of medicinal plants since they are inexpensive and ‎widely available. According to the statics of world health organization, more than 80% of world population ‎particularly in the under- developed countries, provide their primary healthcare necessities from medicinal herbs (W‎HO, 2007 ▶). The history of using medicinal plant to treat diseases goes back to the ancient history. The study of ‎local knowledge about medicinal herbs is becoming increasingly important in defining strategies for conservation ‎and utilization of biological resources (Jeruto et al., 2008 ▶). The notable use and commercialization of medicinal ‎plants to alleviate and cure health problems and ailments in all cities of the country, points out the importance of ‎these natural resources in the folk medicine and culture of the Iranian people (Emami et al., 2012 ▶). However, most ‎of the useful information is still available for traditional healers and knowledge of healers is either lost or passed to ‎next generation by the word of mouth (Yirga, 2010 ▶). In many developing countries, medicinal plants have not been ‎well studied, tested, or documented (Amiri and Joharchi, 2013 ▶). Ethnobotany deals with the collection of valuable ‎medicinal plants by a group of people and describes their different uses (Safa et al., 2012 ▶). Hence, ‎identification of useful medicinal plants is an excellent policy to understand their properties by indigenous ‎inhabitants. Our surrounding nature is the habitat of many unknown medicinal plants that indigenous people use ‎ for treating their ailments. Iran, by having varied climate and geographical regions and also different types of ‎mountains, plains, deserts, hills, river and lakes, and wetlands is considered to be a center for accessing valuable and ‎scare medical species (Ahvazi et al., 2012 ▶). The native knowledge of medicinal plants has been put in danger of ‎being lost by assimilating these tribes and loss of traditional community life (Mosaddegh et al., 2012 ▶). Therefore, it ‎seems necessary to perform ethnobotanical studies in Iran to record all the knowledge of folk medicine practiced ‎among native people (Naghibi et al., 2005 ▶). Arjan – Parishan protected area with two very beautiful wetlands ‎Parishan and Arjan is situated 60 km west of Shiraz in Fars province. This geographic region is one of the ‎most important human migration roads in Iran, showing a great plant biodiversity, so traditional usage of ‎medicinal plant is a familiar therapeutic way for native people. In recent years, traditional use of plants for medical ‎purposes has drawn the attention of researchers in our country as well (Ahvazi et al., 2012 ▶; Mirdeilami et al., 2011 ▶; ‎Ghorbani, 2005 ▶; Mosaddegh et al., 2012 ▶; Safa et al., 2012 ▶). However, there are no published records on ‎ethnobotanical knowledge of medicinal plants in the area.‎

The main objective of the present study was to elicit data on the traditional uses of medicinal plants in the Arjan- ‎Parishan protected area. 

**‎** **Study area**

**Figure 1:**
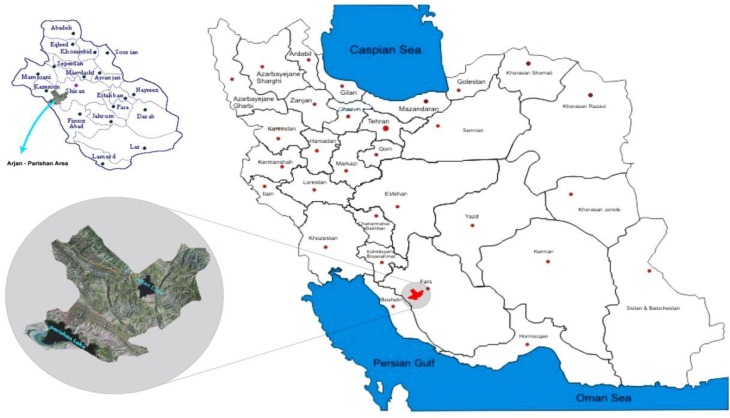
Study area: Geographical location of Arjan- Parishan area in Fars Province, Iran

The Arjan – Parishan protected region (29˚ 34' 48'' N and 51˚ 54' 36'' E) covers an area of about 60000 hectares in ‎southwest Iran (Figure 1), receiving an average annual rainfall of about 430 mm. This very beautiful area of ‎attractive landscape such as the Arjan and Parishan wetlands is located between Kazeroon and Shiraz. The vast ‎majority of the residents of this region are ethnic Persians. In this area, agriculture plays the main economic role.‎

 People of the Arjan – Parishan region have a long history of utilizing medicinal plants to cure their diseases ‎according to their cultural background. This area is important for plant biodiversity due to the presence of some ‎important habitats such as international wetland of Parishan and “oak forest” that are dominated by Quercus ‎brantii L. Approximately, 60 % of this area is surrounded by Zagros Mountain. International Wetland of Parishan ‎is located 12 km to the southeast of Kazeroon. The climate of this area is arid and cold desert with the average ‎elevation 820 mabove sea level. Arjan wetland with altitude of 2015 m above sea level is situated 60 km ‎west of Shiraz in Fars province. This area has semi-arid to semi-humid climate. Due to variation in altitude, ‎topography, and bio-climate within this area, the diversity of medicinal plants and indigenous medical knowledge ‎are rich. Therefore, this biodiversity can be important in aspects of ethnobotanical and pharmaceutical potentials. ‎At present, the Arjan – Parishan area is considered as protected area by IUCN classification.

## Materials and Methods

In order to gather information on medicinal species found in the Arjan- Parishan protected area, an investigation was performed during 2010-2012. According to the geographical and topographic maps, various parts of the region were referred in proper seasons and then the vernacular information of plants and their usages were collected from well-experienced people and finally all collected plants specimens were dried and pressed. All plant species encountered during field observations were recorded. A questionnaire was administered to the local people, through face-to-face interviews. During the interviews, local names, utilized parts, and preparation methods of the plants as well as information on the types of ailments treated using traditional medicinal plant species were recorded. The informants were selected as they were known as being knowledgeable by the local community. Interviews were done at informants’ homes, farms, or medicinal plant markets, after making clear that they are participating in a research project with the purpose of saving the local traditional knowledge. Collected plants were recognized and their families, genera, and species were determined using of Flora Iranica (Assadi et al., 1989 ▶; Awan et al., 2011 ▶; Parsa and Maleki, 1978 ▶; Rechinger, 1987), Flora of Turkey (Davis, 1965-1988 ▶), Flora of Syria (Post and Dinsmore, 1932 ▶), and Flora of Iraq (Townsend et al., 1966-1985 ▶). Identified plants were deposited at the herbarium of Payame Noor university center of Bushehr.

## Results

**Figure 2:**
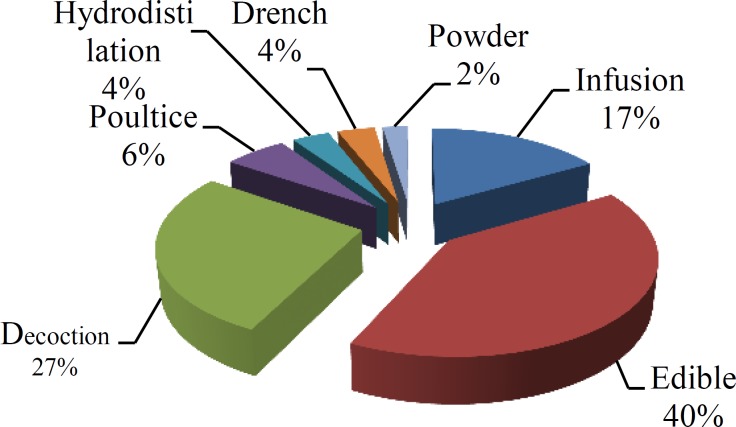
Mode of preparations and their percentages

The present ethnobotanical survey gathered information on 85 plant species reported by the informants for their medicinal use (see Tables 1). The species belonged to 78 genera and 39 families. Collected species included two ‎pteridophyta, one gymnosperma, two monocotyledons, and 80 dicotyledones (the largest order in the medicinal flora of ‎area). According to results of this experiment, the largest genera were Anthemis, Artemisia, Capparis, Morus, Rumex, ‎Ziziphus, and Amygdalus (2 species each). The most common application methods were edible (40%) followed by ‎decoction (27%), infusion (17%), poultice (6%), hydrodistillation (4%), drench (4%), and powder (2%) (Figure 2). ‎

**Table1:** Medicinal plant species collected from Arjan - Parishan protected area and their traditional uses.

**Family**	**Scientific name**	** Local name**	** Part used**	**Local uses**	**Uses**	**H. No**
**Anacardiaceae**	*Pistacia khinjuk *Stocks.	Kolkhong	Fr	Body reinforcement, Joint and muscles pain	Edible, Poultice	522
**Apiaceae**	*Ammi majus *L.	Khelal Dandoon	A. p	As toothpick	Edible	547
	*Anethum graveolens *L.	Sheved	Se, L	Indigestion in children Blood Lipid, Joint pain	Edible	591
	*Coriandrum* *sativum* L.	Gishniz	L, Se, St	Reducing blood lipid and sugar, Flatulency, Antiseptic	Edible, Decoction	609
	*Foeniculum vulgare *Mill.	Rajuoneh	Se	Painful menstruation, Joint pain, Flatulency, Back pain, Nervous weakness	Decoction	565
	*Oliveria decumbens *Vent.	Den	Fr	Stomachache, Dyspepsia, Flatulency	Decoction	624
**Araceae**	*Biarum straussii * Engl.	Kardeh	L	Kidney stone, Cholagogue	Edible	637
**Asteraceae**	*Achillea tenuifolia *Lam.	Bimadaroon	Fr	Blood fat, Flatulency, Abdominal pain	Infusion	502
	*Anthemis altissima* L.	Baboone	Fr	Heart tonic Menstruate pain	Infusion	538
	*Anthemis austroiranica* Rech.f., Aellen & Esfand.	Baboone	Fr	Heart tonic Menstruate pain	Infusion	615
	*Artemisia annua *L.	Dermane	A. p, Fl	Stomachache, Blood fat	Edible	545
	*Artemisia* *dracunculus* L**.**	Tarkhonii	St, L	Decrease blood pressure Appetizing, Spice,	Edible	651
	*Calendula persica* C.A. Mey.	Gole Gorbe	Fl, L	Skin diseases	Decoction	664
	*Centaurea bruguierana *(DC.) Hand.-Mazz.	Bad Bord	Fr	Blood sugar, Diabetes	Infusion	564
	*Cichorium intybus* L.	Kashni	St, L	Liver tonic	Hydrodistilation	521
	*Cynara* *scolymus* L.	Kangar	L, Rh	Cooling	Edible	544
	*Echinops cephalotes* DC.	Shekarook	Re	Digestive problems,	Decoction	537
	*Lactuca serriola *L.	Bikh Bonje	L	Appetizing, Cholagogue	Edible	671
	*Matricaria recutita *L.	Baboone Gawy	Fr	Antimicrobial, Hair tonic	Infusion	511
	*Silybum marianum *(L.) Gaertn.	harKhangaloo	Se, Fl	Decrease blood pressure	Decoction	567
**Berberidaceae**	*Berberis vulgaris *L.	Zereshk	Fr	Fever, Liver tonic, Heart tonic	Edible	641
**Boraginaceae**	*Anchusa italica *Retz.	Gol GoZaboon	Fr	Treatment of respiratory problems	Infusion	536
	*Heliotropium europaeum *L.	Oftow Paras	A. p	Scorpions Bite	Decoction	548
**Brassicaceae**	*Capsella bursa-pastoris* (L.) Medik.		St, Fl	Astringent, Anti blood pressure, Gastrodynia	Drench	593
	*Nasturtium officinale* R. Br.	Bakaloo	ST, L	Kidney stone	Infusion	673
	*Sisymbrium loeselii* L.	Khakshir	Se	Vitamin c content	Drench	596
**Capparidaceae**	*Capparis parviflora* Boiss.	Kewerak-Lagjin	St, Fr, Fl	Flatulency, Diuretic Astringent, Rheumatism	Edible	569
	*Capparis spinosa* L.	Kewerak-Lagjin	St, Fr, Fl	Diuretic, Astringent, Rheumatism, Blood fat and sugar, Hemorrhoids	Edible	519
**Cucurbitaceae**	*Citrullus colocynthis* (L.) Schrad.	Khiar Gorgoo Hendoone Aboo Jal	Se	Diabetes, Laxative	Powder	534
**Cuscutaceae**	*Cuscuta kurdica* Engelm.	Saratan	Wh. p	Depression, Analgesic	Decoction	543
**Ephedraceae**	*Ephedra* *pachyclada* Boiss.	Hoonder	St, L	Mouthwash	Decoction	563
**Equisetaceae**	*Equisetum arvense *L.	Dom Asbi	St, L	Wound washing	Decoction	642
**Euphorbiaceae**	*Euphorbia helioscopi*a L.	Shir Shirook	La	Warts	Poultice	597
	*Ricinus communis* L.	Kernatoo	Se	purgative	Poultice	550
	*Quercus brantii* Lindl.	Bali	Fr	Astringent, Diarrhea	Edible	601
**Fumariaceae**	*Fumaria vaillantii* Loisel.	Shatarreh	Wh.P	Cold	Hydrodistilation	687
**Juglandaceae**	*Juglans regia *L.	Gerdoo	Fr	Treatment of anemia, Improve memory	Edible	551
**Lamiaceae**	*Mentha longifolia *(L.) Huds.	Pidom	Fl, L	Heatstroke, Jaundice	Edible	603
	*Melissa officinalis *L.	Badranjbouie	Se, L	Sedative	Decoction	532
	*Ocimum basilicum *L.	Reihoon	L	Fever, Mouth wound	Edible	562
	*Otostegia persica *(Burm.) Boiss.	Shekar Shafa	Fr	Diabetes	Decoction	542
	*Salvia macrosiphon *Boiss.	Gol Pashe Paroon	A. p	Treatment of respiratory problems, Prevention of insects bite	Edible	643
	*Teucrium polium *L.	Alpe	L, Fl	Regulating blood lipid and Sugar, Diabetes, scented	Infusion	571
	*Vitex agnus-castus *L.	Bangroo	Fl, L, Fr	Astringent,Hemorrhoids	Decoction	675
**Malvaceae**	*Alcea aucheri *(Boiss.) Alef.	Gol Khatmii	L, Fl, R	Emollient, Prevention of hair loss	Infusion	685
	*Malva parviflora *L.	Toolak	L, Fl	Treatment of Kidney and bladder infections, Emollient	Edible	683
**Moraceae**	*Ficus* *carica* L.	Anjir	Fr, La	Purgative, Warts	Edible	605
	*Morus alba *L*.*	Tite Safid	Fr, La	Emollient, Cold	Edible	504
	*Moru*s *nigra *L.	Tite Siah	Fr	Emollient, Cold	Edible	560
**Myrtaceae**	*Myrtus communis *L.	Mourd	L	Fatigue	Vapor	
**Oleaceae**	*Olea europaea *L.	Zeytoon	Fr	Emollient	Edible	541
**Papilionaceae**	*Alhagi camelorum *Fisch*.*	Khar Shotor Toranjabin	Wh.P	Kidney stone	Decoction	573
	*Glycyrrhiza glabra *L*.*	Meik Mahak	R	Pectoralgia, Bone pain, Fatigue	Decoction	531
	*Melilotus indicus *(L.) All.	Shabdar	L, Fl	Blood diluents	Decoction	645
	*Prosopis farcta *(Banks & Sol.) J.F. Macbr.	Kharak Sag	Se	Healing the wounds	Poultice	559
	*Trifolium repens *L.	Shabdar		Expectorant, Emmenagogue		590
**Plantaginaceae**	*Plantago major* L.	Barhang	Se	Expectorant, Emollient, Pectoralgia	Infusion	606
**Poaceae**	*Phragmites australis* (Cav) Trin.ex.Steud	Ney	L, Fl	Stopping mother milk	Decoction	676
**Podophyllaceae**	*Leontice leontopetalum* L.	Tegh Tegh Konak	Fr	Pectoralgia	Decoction	574
**Polygonaceae**	*Rumex* *dentatus* L.	Torshook	L	Appetizing, Cholagogue	Edible	646
	*Rumex vesicarius* L.	Torshook	L	Appetizing, Cholagogue	Edible	552
**Portulacaceae**	*Portulaca oleracea* L.	Ghorfe	L, Se	Diuretic, Blood purifier, Anti blood lipid	Edible	517
**Primulaceae**	*Anagalis arvensis *L.	Anaghalis	Fr	Insect bites, Diuretic, Expectorant	Oultice	558
**Pteridaceae**	*Adiantum Capillus-Veneris *L.	Parsiavashoon	L	Earache, Common cold, Kidney stones Expectorant	Decoction Infusion	510
**Punicaceae**	*Punica granatum *L.	Anar	Fr	Appetizing, Jaundice, Cholagogue	Edible	505
**Ranunculaceae**	*Adonis dentata *Delile.	Gole Atashin	L , Fl	Rheumatism	Decoction	579
**Rhamnaceae**	*Ziziphus nummularia* (Burm. f.) Wight & Arn.	Lamrik	Fr	Appetizing, Cholagogue	Edible	586
	*Ziziphus spina-christi* (L.) Willd.	Konar	L, Fr	Washing hair, Cold	Powder, Edible	607
**Rosaceae**	*Amygdalus communis *L.	Badoum	Fr	Skin diseases, Treatment of asthma	Infusion	540
	*Amygdalus glauca *Browicz.	Akhorak	Fl, Se	Skin diseases, treatment of asthma	Infusion	516
	*Crataegus aronia *(L.) Bosc. ex Dc.	Kiial	Fr	Insomnia- migraine Cholagogue,	Edible	680
	*Malus communis *Desf.	Sib-E Kohi	Se	Vitamin, Tonic	Edible	530
	*Pyrus communis *L.	Anchochak	Se	Kidney stone	Edible	580
	*Rubus sanctus* Schreb.	Tit Are	Fr	Diuretic, Appetizing, Expectorant	Edible	584
**Salicaceae**	*Salix alba *L*.*	Bidmeshk	Ba	Rheumatism	Hydrodistilation	608
**Solanaceae**	*Datura stramonium *L*.*	Tatureh	Se, L	Gout, Burning wounds		556
	*Hyoscyamus tenuicaulis* Schönb.-Tem.	Bang Done	Se	Anti- asthma, Sedative		682
	*Solanum nigrum *L.	Rob Torwak	Fr	Emollient, Reducing blood lipid and glucose, Bronchitis, Pectoralgia	Decoction	648
**Urticaceae**	*Urtica pilulifera* L.	Gazane	Wh.p	Rheumatism, Rash	Decoction	506
**Verbenaceae**	*Verbena officinalis* L	Shapasand	A. p	Blood purifier, Fever	Decoction	527
**Vitaceae**	*Vitis vinifera *L.	Angour	Fr	Appetizing, Contain a variety of vitamins	Edible	553
**Zygophylaceae**	*Peganum harmala* L.	Dounesht	Fr	Rheumatism, Antiseptic, Expectorant	Decoction	524
	*Tribulus terrestris* L.	Khar Pelangi	Wh. p	Kidney stone	Infusion	514

**Figure 3:**
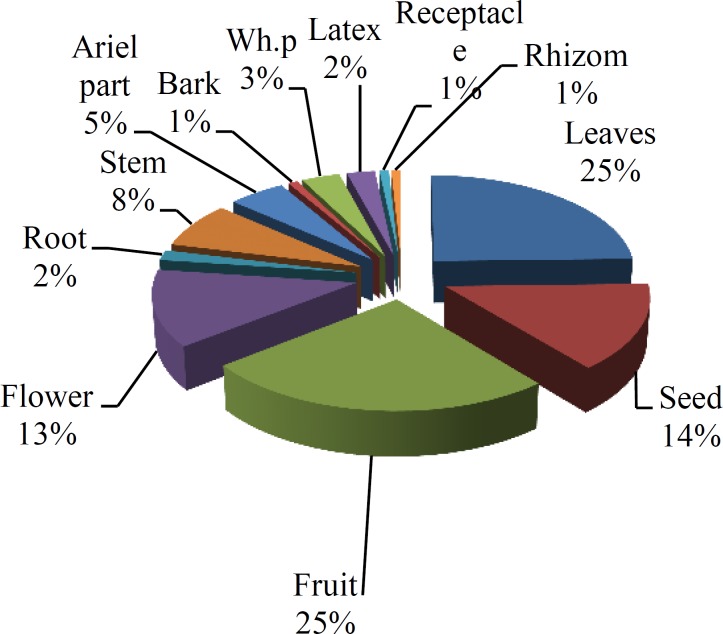
Plant parts used in treating ailments and their percentage

**Figure 4:**
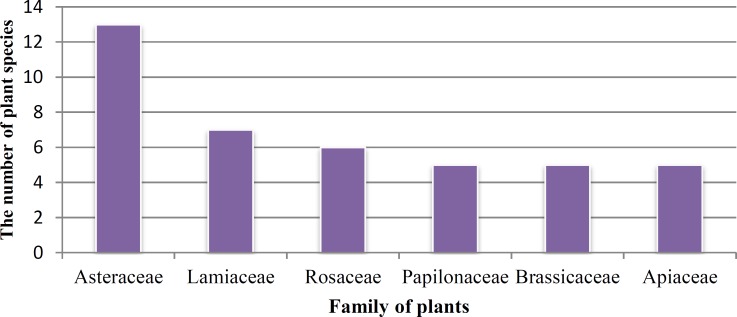
Plant families with the highest number of cited species

However, some plants were used in more than one method of preparation. Different parts of medicinal plants ‎‎(roots, leaves, fruits and seeds, intact plant, etc.) were used by the local inhabitants as medicines (23). Fruits and ‎leaves each with (25%) followed by seeds (14%), flowers (13%), stem (8%), aerial parts (5%), whole plant (3%), ‎latex (2%), root (2%), rhizome (1%), receptacle, (1%) and bark (1%) were among the most widely used medicinal ‎parts (Figure 3). In this paper, we also mentioned the list of most efficient medicinal plants of the Arjan - Parishan ‎protected area for treating ailments (Table 2). As shown in Figure 4, Asteraceae with 13 species followed by ‎Lamiaceae with seven species, Rosacesae with six species and Apiaceae, Brassicaceae, and Papilionaceae families each ‎with five species were the most frequent families in the area.

**Figure 5:**
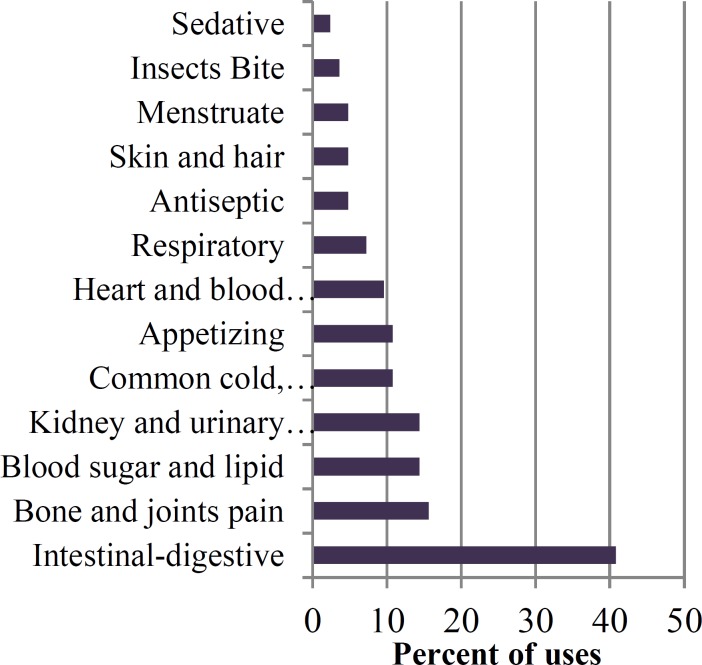
Ailments treated by medicinal plants along with their percent

The results obtained from the present study indicated ‎that medicinal plants of the Arjan – Parishan protected area are used in the treatment of many diseases particularly ‎for intestinal-digestive disorders (40.8%), bone and joints pain (15.6%), kidney and urogenital diseases (14.4%), ‎blood sugar and lipid (14.4%), common cold, expectorant, and fever (10.8%), appetizing (10.8%), heart-blood ‎circulatory system disorders (9.6%), respiratory disorders (7.2%), antiseptic (4.8%), skin and hair (4.8%), menstruate ‎‎(4.8%), insect bite (3.6%), and sedative (2.4%) (Figure 5).‎

## Discussion

During recent decades, chemical side effects have been identified and actions have been taken to overcome this ‎problem (Mozaffari Nejad et al., 2013 ▶). Hence, even in the modern age, in developed countries, people still rely on ‎traditional system of healthcare not only because of its low price, but also due to low side effects as compared to ‎modern allopathic medicine (Awan et al., 2011 ▶). It is believed that rational use of native medicinal plants along ‎with effective synthetic drugs may benefit and improve the quality of life and living standards of the native ‎inhabitants (Namsa et al., 2011 ▶; Oliveira et al., 2011 ▶). Despite the importance of these plants for health ‎improvement, it seems that some of the most promising medicinal plants have not yet been fully identified. For this ‎reason, documentation of the indigenous knowledge through ethno- botanical studies is important for the ‎conservation and utilization of biological resources (Muthu et al., 2006 ▶).‎ ‎ Because of seasonal, soil, climatic, and topography variation, Iran is rich in plant biodiversity and especially ‎medicinal plants. 

**Table 2:** The most efficient medicinal plants of Arjan - Parishan protected area for treating ailments

**Digestive**	Foeniculum vulgare, Oliveria decumbens, Biarum straussii, Achillea tenuifolia, Artemisia annua, Artemisia dracunculus, Cichorium intybus, Echinops cephalotes, Lactuca serriola, Berberis vulgaris, Capsella bursa-pastoris, Cardaria draba, Descurania Sophia, Capparis parviflora, Capparis spinosa, Citrullus colocynthis, Ricinus communis, Quercus brantii, Mentha longifolia, Vitex agnus-castus, Alcea aucheri, Malva parviflora, Ficus carica, Morus alba, Morus nigra, Olea europaea, Glycyrrhiza glabra, Plantago major, Rumex dentatus, Rumex vesicarius, Punica granatum, Crataegus aronia, Rubus sanctus, Datura stramonium, Solanum nigrum, Vitis vinifera
**Kidney and Urinary system**	Adianthum capillus-veneris, Biarum straussii, Nasturtium officinale, Capparis parviflora, Capparis spinosa, Malva parviflora, Alhagi camelorum, Portulaca oleracea, Anagalis arvensis, Rubus sanctus, Hyoscyamus tenuicaulis, Tribulus terrestris
**Heart and blood vessels**	Anthemis altissima, Anthemis austro-iranica, Artemisia dracunculus, Silybum marianum, Berberis vulgaris, Capsella bursa-pastoris, Juglans regia, Melilotus indicus, Prosopis farcta, Portulaca oleracea, Verbena officinalis
**Skin and Hair**	Calendula pérsica, Matricaria recutita, Euphorbia helioscopia, Alcea aucheri, Ficus carica, Morus alba, Ziziphus nummularia, Ziziphus spina-christi, Amygdalus communis, Amygdalus glauca, Urtica pilulifera
**Respiratory**	Anchusa italic, Equisetum arvense, Salvia macrosiphon, Glycyrrhiza glabra, Plantago major, Amygdalus communis, Amygdalus glauca, Datura stramonium, Hyoscyamus tenuicaulis, Solanum nigrum
**Common Cold, Antipyretic And ** **Expectorant**	Adiantum capillus-veneris L., Berberis vulgaris, Ocimum basilicum, Trifolium repens, Plantago major, Anagalis arvensis, Rubus sanctus, Verbena officinalis, Peganum harmala
**Blood Sugar**	Centaurea bruguierana, Capparis spinosa, Citrullus colocynthis, Otostegia persica, Teucrium polium, Solanum nigrum
**Blood Lipid**	Anethum graveolens, Achillea tenuifolia, Artemisia annua, Teucrium polium, Portulaca oleracea, Solanum nigrum
**Rheumatism**	Capparis parviflora, Capparis spinosa, Adonis dentate, Salix alba, Urtica pilulifera, Peganum harmala
**Depression and ** **Nerve system relaxant**	Cuscuta kurdica, Melissa officinalis, Crataegus aronia, Salix alba, Hyoscyamus tenuicaulis
**Mouth** **and Tooth**	Ammi majus,Matricaria recutita, Ephedra pachyclada, Juglans regia, Ocimum basilicum
**Antiseptic**	Centaurea bruguierana, Matricaria recutita, Descurania Sophia, Peganum harmala
**Bone, Joints and Muscle**	Pistacia khinjuk, Capparis spinosa, Myrtus communis, Glycyrrhiza glabra
**Reconstituent and Vitminae**	Pistacia khinjuk, Sisymbrium loeselii, Capparis spinosa
**Menstruate**	Anthemis altissima, Anthemis austro-iranica, Trifolium repens
**Insects Bite**	Heliotropium europaeum, Salvia macrosiphon

The Arjan – Parishan protected area comprise great biodiversity of plant species bearing variation ‎of climatic and also different ecological habitats such as mountains, hills, plains, valleys, and lakes. It appears that ‎there are many medicinal uses for the treatment of different diseases in the study area which were rarely revealed ‎before this. According to the current study, Asteraceae and Lamiaceae were the dominant locally used families (Figure 4). Our results are also in ‎agreement with ethnobotanical studies performed in other parts of Iran such as Hormozgan province (Safa et al., ‎‎2012 ▶), Kohghiluyeh va Boyer Ahmad province (Mosaddegh et al., 2012 ▶), and Maraveh Tappe region, north of Iran ‎‎(Mirdeilami et al., 2011 ▶). It may be due to adaptation of these families with arid and semiarid conditions. ‎Moreover, from the large genera found in this area, Ziziphus and Amygdalus can be referred which provide ‎suitable habitat for other medicinal plants because of the vicinity to the Zagros mountain range. From the 85 species ‎reported in this paper, some of the plants are being used more frequently and also are well-known compared to others ‎which may be due to their availability and knowledge of the local people.Among them, Adiantum capillus- veneris L., ‎Oliveria decumbens, Achillea tenuifolia, Anthemis altissima, Anthemis austro-iranica, Cynara scolymus, Berberis ‎vulgaris, Nasturtium officinale, Capparis spinosa,‎‏ ‏Citrullus colocynthis, Quercus brantii, Melissa officinalis, ‎Ocimum basilicum, Teucrium polium,‎‏ ‏Malva parviflora, Ficus carica, Olea europaea, Alhagi camelorum, ‎Plantago major, and Portulaca oleracea can be named. Some of medicinal plants in this region belong to different species of ‎a genus, but their species are all known to one local name. The best examples are ‎Anthemis austro-iranica, Anthemis altissima,‎‏ ‏Rumex dentatus, Rumex vesicarius, Capparis spinosa, and Capparis ‎parviflora. Some other medicinal plants in this region have vast distribution such as Pistacia ‎khinjuk, Achillea tenuifolia, Capparis spinosa, Euphorbia helioscopia, Mentha longifolia, and Olea europaea. Among ‎these medicinal plants, some are located in impossible places such as Pistacia khinjuk, Crataegus aronia, Malus ‎communis, Pyrus communis, therefore, they are used mostly by native people who have easier access to them. ‏.‏In addition, some ‎plants have both medicinal and edible uses and increasingly entered the market in specific seasons, ‎such as Cynara scolymus, Berberis vulgaris, Juglans regia, Ficus carica, Olea europaea, Vitis vinifera, Crataegus ‎aronia, Punica granatum, and Ocimum basilicum. It seems that there are many medicinal uses for the treatment of ‎several ailments and illnesses in the studied area. Traditional understanding of phytotherapy of this district provides ‎excellent outcome in treating different types of ailment such as intestinal-digestive disorders, followed by bone and ‎joint pain, kidney and urogenital diseases, blood sugar and lipid, common cold, expectorant and fever, appetizing, ‎heart-blood circulatory system disorders, respiratory disorders, antiseptic, skin and hair, menstruate, insect bite, as well as as a ‎sedative. The high use of medicinal plants by the native inhabitants to cure intestinal-digestive ailments could be ‎attributed to the high preponderance of these disorders in the area. It appears that the gastrointestinal system is the ‎most common use in studies in different districts of Iran (Mosaddegh et al., 2012 ▶; Miraldi et al., 2001 ▶). The most ‎frequently used parts by local people were leaves and fruits. Our data are in agreement with the recent results of ‎Rajaei and Mohamadi (2012) ▶. They reported that the leaves are the dominant part used. As shown in Table 2, in order to ‎relieve pain, people use some plants that are mentioned more frequently by the informants for the same use compared to other plants ‎such as Glycyrrhiza glabra L, Pistacia khinjuk, Capparis spinosa, and Myrtus communis. Considering the extreme ‎importance of plants of the area in treating gastrointestinal ailments, it is recommended to conduct further studies to ‎identify the active ingredients of these herbs.‎

In this research paper, efforts have been made to document the traditional knowledge of important medicinal ‎plants of the Arjan – Parishan protected area. The presence of 85 medicinal plants indicates high biodiversity of ‎medicinal plant in the region. These plants are abundantly found in this region and are considered to be ‎used for treatment of various diseases. It is concluded that the Arjan – Parishan protected area has good ‎ethnobotanical potentials for medicinal plants and all of the plants found in this study are most favorite among the local ‎people. According to the results of this research, fruits and leaves are the major used parts in this region. It ‎is important to emphasize that intestinal-digestive system is the first target for traditional medicine in the area. ‎Therefore, the information documented on the medicinal plants of the Arjan – Parishan protected area may serve ‎as baseline data for future pharmacological and phytochemical studies and consequently discover new drugs.‎

## Conflict of interest

We certify that there is no conflict of interest with any financial organization regarding the manuscript.

## References

[B1] Ahvazi M, Khalighi-Sigaroodi F, Charkhchiyan MM, Mojab F, Mozaffarian V, Zakeri H (2012). ‎Introduction of medicinal plants species with the most traditional usage in Alamut region. Iran J Pharm Res.

[B2] Amiri MS, Joharchi MR (2013). Ethnobotanical investigation of traditional medicinal plants ‎commercialized in the markets of Mashhad, Iran. AJP.

[B3] Assadi M, Maassoumi AA, Khatamsaz M, Mozaffarian VA (1990-2010). Flora of Iran. Research‎‏ ‏Institute ‎of Forests and Rangeland.

[B4] Awan MR, Iqbal Z, Shah SM, Jamal Z, Jan G, Afzal M, Majid A, Gul A (2011). Studies on traditional ‎knowledge of economically important plants of Kaghan Valley, Mansehra district, Pakistan. J Med Plants Res.

[B5] Davidson-Hunt I (2000). Ecological ethnobotany: stumbling toward new practices and paradigms. MASA J.

[B6] Davis PH (1965- 1988). Flora of Turkey and the East Aegean Islands.

[B7] Emami SA, Nadjafi F, Amine GH, Amiri MS, Khosravi MT, Nasseri M (2012). Les espèces de plantes ‎médicinales utilisées par les guérisseurs traditionnels dans la province de Khorasan, nord-est de l'Iran. J ‎Ethnopharmacol.

[B8] Ghorbani A (2005). Studies on pharmaceutical ethnobotany in the region of Turkmen Sahra, north of Iran ‎‎(Part 1): General results. J Ethnopharmacol.

[B9] Jeruto P, Lukhoba C, Ouma G, Otieno D, Mutai C (2008). An ethnobotanical study of medicinal plants used ‎by the Nandi people in Kenya. J Ethnopharmacol.

[B10] Miraldi E, Ferri S, Mostaghimi V (2001). Botanical drugs and preparations in the traditional medicine of ‎west Azerbaijan (Iran). J Ethnopharmacol.

[B11] Mirdeilami SZ, Barani H, Mazandarani M, Heshmati GA (2011). Ethnopharmacological survey of ‎medicinal plants in Maraveh Tappe region, north of Iran. Iran J Plant Physiol.

[B12] Mosaddegh M, Naghibi F, Moazzeni H, Pirani A, Esmaeili S (2012). Ethnobotanical survey of herbal ‎remedies traditionally used in Kohghiluyeh va Boyer Ahmad province of Iran. J Ethnopharmacol.

[B13] Mozaffari Nejad AS, Kamkar A, Giri A, Pourmahmoudi AA (2013). Ethnobotany and folk medicinal uses ‎of major trees and shrubs in northern Iran. J Med Plants Res.

[B14] Muthu C, Ayyanar M, Raja N, Ignacimuthu S (2006). Medicinal plants used by traditional healers in Kancheepuram ‎district of Tamil Nadu, India. J Ethnobiol Ethnomed.

[B15] Naghibi F, Mosaddegh M, Mohammadi Motamed S, Ghorbani A (2005). ‎‏ ‏Labiatae family in folk medicine ‎in Iran: from ethnobotany to pharmacology. Iran J Pharm Res.

[B16] Namsa ND, Mandal M, Tangjang S, Mandal SC (2011). Ethnobotany of the Monpa ethnic group at ‎Arunachal Pradesh, India. J Ethnobiol Ethnomed.

[B17] ‎ Oliveira AKM, Oliveira NA, Resende UM, Martins PFRB (2011). Ethnobotany and traditional medicine of ‎the inhabitants of the Pantanal Negro sub-region and the raizeiros of Miranda and Aquidauna, Mato Grosso do Sul, ‎Brazil. Brazil J Biol.

[B18] Parsa A (1978-1980). Flora of Iran.

[B19] Post GE, Dinsmore JE (1932). Flora of Syria, Palestine and Sinai.

[B20] Rajaei P, Mohamadi, N (2012). Ethnobotanical study of medicinal plants of Hezar mountain allocated in ‎south east of Iran.. Iran J Pharm Res.

[B21] Rechinger KH (1963-2005). Flora Iranica.

[B22] Safa O, Soltanipoor MA, Rastegar S, Kazemi M, Nourbakhsh Dehkordi Kh, Ghannadi A (2012). An ‎ethnobotanical survey on Hormozgan province, Iran. AJP.

[B23] Townsend CC, Guest E, Al-rawi A (1966-1985). Flora of Iraq.

[B24] World Health Organization (2007). WHO monographs on selected medicinal plants.

[B25] Yirga G (2010). Assessment of traditional medicinal plants in Endrta district, southeastern Tigray, northern ‎Ethiopia. J Med Plants Res.

